# A model to predict red blood cell transfusion during/after cesarean delivery

**DOI:** 10.1002/pmf2.70249

**Published:** 2026-02-24

**Authors:** Samuel Parry, Steven J. Weiner, George R. Saade, Rebecca G. Clifton, John M. Thorp, Monica Longo, Ashley Salazar, Wendy Dalton, Alan T. N. Tita, Cynthia Gyamfi-Bannerman, Suneet P. Chauhan, Torri D. Metz, Kara Rood, Dwight J. Rouse, Jennifer L. Bailit, William A. Grobman, Hyagriv N. Simhan, George A. Macones, Luis D. Pacheco

**Affiliations:** 1 Department of Obstetrics and Gynecology, Perelman School of Medicine at the University of Pennsylvania, Philadelphia, Pennsylvania, USA; 2 Department of Epidemiology, The George Washington University Biostatistics Center, Washington, District of Columbia, USA; 3 Department of Obstetrics and Gynecology, The University of Texas Medical Branch, Galveston, Texas, USA; 4 Department of Obstetrics and Gynecology, The University of North Carolina at Chapel Hill, Chapel Hill, North Carolina, USA; 5 The Eunice Kennedy Shriver National Institute of Child Health and Human Development, Bethesda, Maryland, USA; 6 Department of Obstetrics and Gynecology, Case Western Reserve University, Cleveland, Ohio, USA; 7 Department of Obstetrics and Gynecology, The University of Alabama at Birmingham, Birmingham, Alabama, USA; 8 Department of Obstetrics and Gynecology, Columbia University, New York, New York, USA; 9 Department of Obstetrics and Gynecology, The Texas Health Science Center at Houston, Children’s Memorial Hermann Hospital, Houston, Texas, USA; 10 Department of Obstetrics and Gynecology, University of Utah Health Sciences Center, Salt Lake City, Utah, USA; 11 Department of Obstetrics and Gynecology, The Ohio State University, Columbus, Ohio, USA; 12 Department of Obstetrics and Gynecology, Brown University, Providence, Rhode Island, USA; 13 Department of Obstetrics and Gynecology, Northwestern University, Chicago, Illinois, USA; 14 Department of Obstetrics and Gynecology, The University of Pittsburgh, Pittsburgh, Pennsylvania, USA; 15 Department of Obstetrics and Gynecology, The University of Texas at Austin, Austin, Texas, USA

**Keywords:** cesarean delivery, postpartum hemorrhage, prediction model, transfusion

## Abstract

**Background:**

Cesarean delivery is a major risk factor for postpartum hemorrhage. A priority for postpartum hemorrhage safety bundles is the assessment of hemorrhage and transfusion risk following cesarean delivery and the identification of patients at increased risk for postpartum hemorrhage requiring red blood cell transfusion. A prediction model based on antenatal and intrapartum risk factors could be used in a standardized manner among all patients when considering crossmatch or other preparatory measures prior to cesarean delivery.

**Objective:**

To develop a multivariable prediction model for red blood cell transfusion associated with cesarean delivery using a prospective contemporary cohort.

**Methods:**

Secondary analysis of a multicenter trial of tranexamic acid (TXA) versus placebo for the prevention of red blood cell transfusion after cesarean delivery was performed. The primary outcome for this analysis was transfusion of packed red blood cells by hospital discharge or 7 days postpartum, whichever came first. Maternal characteristics and risk factors related to labor were compared between patients who received a transfusion and those who did not. Any of the characteristics that differed with a *p* value <0.05 were considered in a multivariable model. Model selection and internal validation were performed using *k*-fold cross-validation. Using backward elimination, variables that remained significant at *p* < 0.05 were retained in each model. The final model was chosen based on the highest area under the receiver operating characteristic curve (ROC) in the validation set.

**Results:**

A total of 418 of 10,961 patients (3.8%) received a red blood cell transfusion. Factors associated with transfusion and retained in the final model included gestational age at delivery <34 weeks or ≥41 weeks, chorioamnionitis, aspirin use in the week before delivery, preoperative hemoglobin level, preoperative platelet count <100,000, oxytocin duration, and cesarean delivery during the second stage of labor. The final multivariable model had excellent discrimination with an area under the curve (AUC) of 0.79 (95% confidence interval [CI], 0.76, 0.81). Using the same set of seven factors identified in the prediction model for transfusion, the AUC was 0.62 (95% CI, 0.60, 0.65) for differentiating patients who did and did not require surgical/radiological interventions in response to bleeding.

**Conclusion:**

We identified several factors associated with risk of red blood cell transfusion during/after cesarean delivery and a model with excellent predictive ability. Risk factors in this model, including lower preoperative hemoglobin level, aspirin use <7 days before delivery, and delivery during the second stage of labor, may be used to risk-stratify patients for preparation for transfusion to reduce postoperative complications that result from bleeding.

## 1 | INTRODUCTION

Postpartum hemorrhage is a leading cause of maternal morbidity and mortality, accounting for approximately 20% of maternal deaths in developing countries and 10% of maternal deaths in the United States [1, 2]. The incidence of postpartum hemorrhage is three to four times greater following cesarean delivery compared to vaginal delivery [3, 4], and as the rate of cesarean delivery has increased, the rate of postpartum hemorrhage has increased as well, almost doubling in the United States over the past 20 years [5]. Therefore, one of the major priorities in postpartum hemorrhage safety bundles is the assessment of hemorrhage and transfusion risk following cesarean delivery and the identification of patients at increased risk for postpartum hemorrhage requiring red blood cell transfusion.

Several prediction models have been developed to identify patients at risk for postpartum hemorrhage and/or transfusion [6–10]. These models use various risk factors, have differing levels of accuracy, and may not be applicable in all clinical settings. Previous work from the Maternal-Fetal Medicine Units Network of the *Eunice Kennedy Shriver* National Institute of Child Health and Human Development (NICHD) used a regression model incorporating variables available at the time of delivery in a cesarean registry [11] and compared established prediction models for transfusion during cesarean delivery admission [12]. Overall, previous studies demonstrate the potential to create a prediction model that could be used in a standardized manner among all patients when considering crossmatch or other preventive measures prior to cesarean delivery. Our objective was to develop a multivariable prediction model for red blood cell transfusion associated with cesarean delivery based on both antenatal and intrapartum risk factors in a large, contemporary, prospective, multicenter cohort, and using statistical methods that are easily interpreted by most readers.

## 2 | MATERIALS AND METHODS

This is a secondary analysis of a multicenter, clinical trial that compared rates of maternal death or red blood cell transfusion among patients undergoing cesarean delivery who were randomized to treatment with one-gram tranexamic acid (TXA) intravenous immediately following umbilical cord clamping versus placebo [13]. The double-blinded trial, which was conducted at 31 hospitals participating in the Maternal-Fetal Medicine Units Network of the NICHD, found that prophylactic administration of TXA during cesarean delivery did not reduce maternal death or the use of packed red blood cell transfusion. Hence, participants in both arms of the clinical trial (*N* = 10,993 total participants) were included in this secondary analysis.

Patients were eligible for the clinical trial if they had a scheduled or unscheduled cesarean delivery of a singleton or twin gestation. Exclusion criteria were maternal age less than 18 years, transfusion or planned transfusion of any blood products during the current admission, contraindications to TXA (e.g., hypersensitivity to TXA or its ingredients, history of seizure disorder, kidney disease, thromboembolic disease, or medical conditions or use of treatments that convey a high risk of thrombosis), patient refusal of blood products, or a plan to administer prophylactic antifibrinolytics such as open-label TXA or uterotonics other than oxytocin postoperatively. Patients who had vaginal deliveries and patients with placenta accreta spectrum (which is known to be associated with a high chance of transfusion) were excluded from this secondary analysis, but patients with known/suspected placenta previa were included.

Trained and certified research staff members, blinded to trial treatment assignment, abstracted information from medical records, including demographics, medical history, and outcomes data. The primary outcome that we considered for the prediction model was transfusion of packed red blood cells by hospital discharge or 7 days postpartum, whichever came first. The secondary outcome was surgical or radiologic procedures to manage postpartum hemorrhage (e.g., laparotomy, evacuation of hematoma, hysterectomy, uterine packing, intrauterine balloon tamponade, or interventional radiologic procedures).

Exposures (demographic and clinical variables available prior to the operating room) that we considered for inclusion in the prediction model were number of fetuses, indication for cesarean, pregnancy type (with or without infertility treatment), gestational age, maternal age, smoking status, liver disease, diabetes, neurologic disease, cardiovascular disease, respiratory disease, renal disease, body-mass index (BMI), third trimester bleeding, polyhydramnios, gestational hypertension/preeclampsia, clinical chorioamnionitis, coagulopathy, preoperative anticoagulation (unfractionated or low-molecular weight heparin, therapeutic or prophylactic doses), low-dose aspirin use, most recent hemoglobin level and platelet count, type of labor, each cervical ripening agent used, oxytocin used, length of time receiving oxytocin, maximum dose of oxytocin, magnesium sulfate used, reached complete cervical dilation, length of labor if labored, number of prior vaginal deliveries, number of prior cesarean deliveries, and trial treatment assignment.

Maternal characteristics and risk factors related to labor were compared between patients who received a transfusion and those who did not. Categorization of some continuous measures was done to simplify the interpretation, based on thresholds known to be related to the risk for hemorrhage. Any of the characteristics that differed with a *p*-value < 0.05 were considered in a multivariable model, with trial treatment assignment always retained. Model selection and internal validation were performed using *k*-fold cross-validation, with *k* = 5. The study cohort was randomly divided into five roughly equal-sized parts. Using backward elimination, a logistic regression model was chosen for each subset of four of the five parts. Variables that remained significant at *p* < 0.05 were retained in each model. The remaining 1/5 of the cohort was used for validation. The final model was chosen based on the highest area under the receiver operating characteristic curve (ROC) in the validation set, with the additional requirement that variables were present in at least four of the five models. Model calibration was assessed graphically. Individual predicted probabilities for transfusion based on the final prediction model were calculated using selected combinations of patient characteristics.

A logistic regression model for the secondary outcome of surgical or radiologic procedures to manage postpartum hemorrhage was fit using the same variables of the final model identified for transfusion.

No imputation for missing values was performed. *p* < 0.05 was used to define statistical significance, and all tests were two tailed. Analyses were performed with SAS 9.4.

## 3 | RESULTS

A total of 10,995 patients (5525 in the TXA group and 5470 in the placebo group) were compared in the clinical trial [13], and 10,961 were included in this secondary analysis (32 patients diagnosed with placenta accreta and two who had vaginal deliveries were excluded). Overall, 44% of patients underwent primary cesarean delivery, and 37% of cesarean deliveries were preceded by labor. Among these 10,961 patients, 418 (3.8%) received a red blood cell transfusion. The median number of units transfused was 2, and 35 patients received more than three units. Thirty-six potential risk factors for transfusion were compared between patients who did and who did not receive a red blood cell transfusion (Table 1). Twenty-one of these potential risk factors were different with a *p* value less than 0.05, and were considered in the multivariable model along with trial treatment assignment. After performing backward elimination for each of the five folds, seven risk factors for transfusion remained significant at *p* < 0.05 and were retained in at least four of the five prediction models. Antenatal factors associated with a significantly greater risk for transfusion were gestational age at delivery <34 and ≥41 weeks, aspirin use in the week before delivery (compared to no aspirin use during the pregnancy or aspirin use but with discontinuation more than a week prior to delivery), lower preoperative hemoglobin, and preoperative platelet count <100,000 (Table 2). Intrapartum factors associated with a significantly greater risk for transfusion were chorioamnionitis, longer oxytocin duration, and complete cervical dilation (cesarean delivery performed in second stage of labor; Table 2).

**FIGURE 1:**
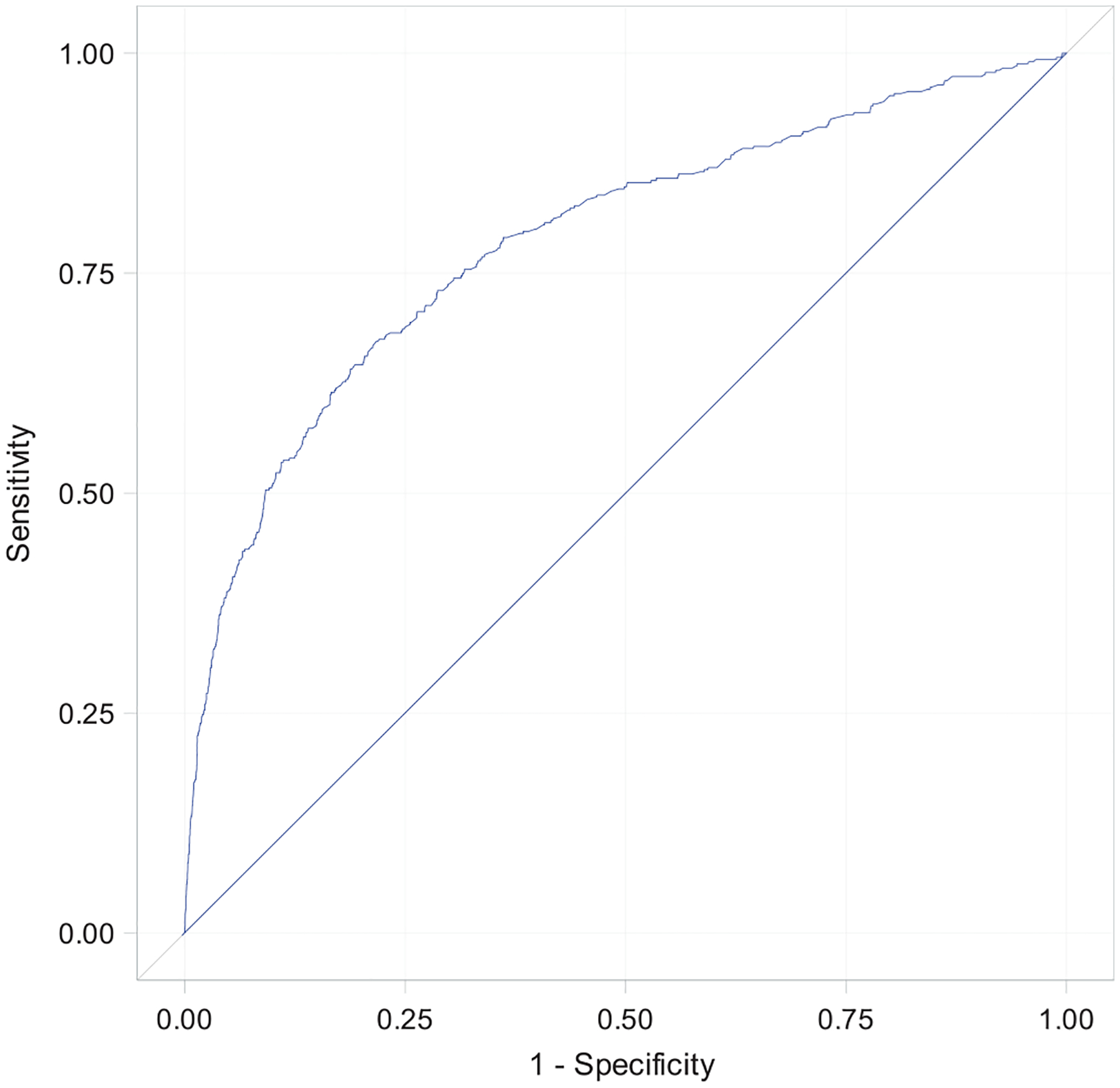
Receiver operator characteristic curve of model validation (transfusion of packed red blood cells).

**FIGURE 2:**
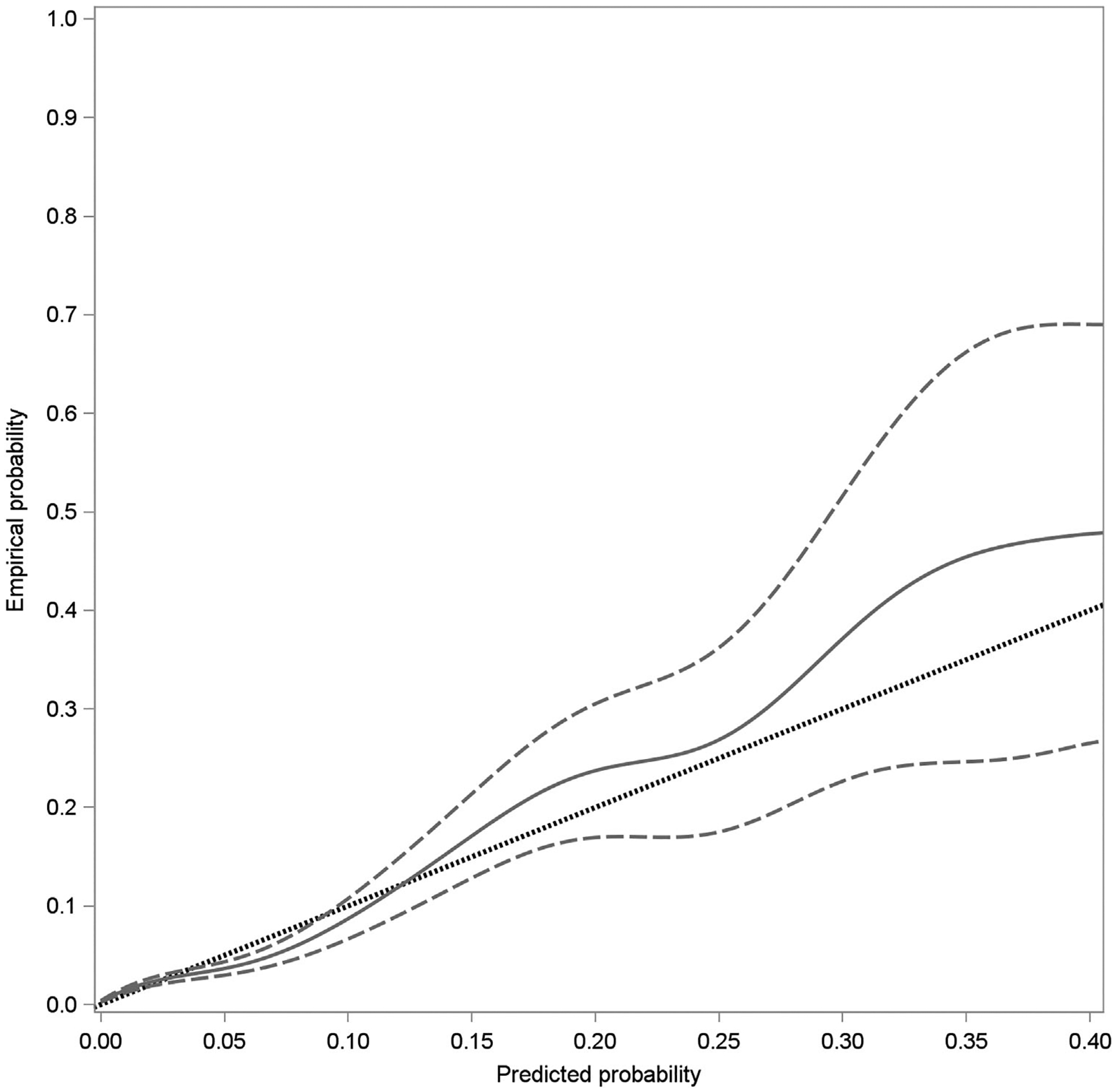
Calibration curve (with 95% confidence interval) of the model validation in the test cohort. The black dashed reference line represents equal predicted and empirical probabilities. The gray solid line represents the point estimate and the gray dashed lines represent the upper and lower 95% confidence limits.

The final multivariate prediction model using the four antenatal and three intrapartum risk factors yielded an ROC that had excellent discrimination with an area under the curve (AUC) of 0.79 (95% confidence interval [CI], 0.76, 0.81; Figure 1). The related calibration results with the estimated curve and its 95% confidence band confirmed that the predicted probabilities for transfusion were overall consistent with the empirical probabilities (Figure 2). Examples of individual predicted probabilities for transfusion based on the final prediction model are listed in Table 3.

Surgical/radiological interventions in response to bleeding occurred in 446 individuals (4.1%, laparotomy, evacuation of hematoma, hysterectomy, uterine packing, intrauterine balloon tamponade, uterine compression sutures, artery ligation, interventional radiology), with 127 of these individuals (28.5%) receiving transfusion. Using the same set of seven factors identified in the final prediction model for transfusion, the AUC was 0.62 (95% CI, 0.60, 0.65) for differentiating those with and without surgical interventions.

## 4 | DISCUSSION

### 4.1 | Principal findings

We developed a multivariable prediction model for red blood cell transfusion associated with cesarean delivery with excellent predictive ability (AUC, 0.79; 95% CI, 0.76, 0.81) in a large, contemporary, prospective, multicenter cohort. The model utilized both antenatal and intrapartum risk factors, including gestational age at delivery <34 and ≥41weeks, aspirin use in the week before delivery (compared to no aspirin use during the pregnancy or aspirin use but with discontinuation more than a week prior to delivery), lower preoperative hemoglobin, and preoperative platelet count <100,000, chorioamnionitis, longer oxytocin duration, and complete cervical dilation (cesarean delivery performed in second stage of labor). Using the same variables, the AUC was 0.62 (95% CI, 0.60, 0.65) for surgical/radiological interventions in response to bleeding (secondary outcome).

### 4.2 | Results in the context of what is known

Most prediction models have used cesarean delivery as a risk for postpartum hemorrhage/transfusion among parturients across all modes of delivery rather than focusing exclusively on patients who have cesarean deliveries [6, 8, 14]. Prediction models focused on cesarean deliveries have clinical importance because the incidence of postpartum hemorrhage is three to four times greater following cesarean delivery compared to vaginal delivery [3, 4], and the rate of cesarean delivery continues to increase.

Recent studies have incorporated machine learning techniques to identify patients at risk for postpartum hemorrhage, and models limited to data available before delivery performed nearly as well as those with more complete data sets, supporting their potential utility in the clinical setting [9, 10]. Further work is needed to create successful models using machine learning techniques based on mode of delivery.

A recent systematic review of 14 prognostic models for postpartum hemorrhage risk found that three clinical scenarios yield potentially useful models—cesarean delivery, placenta accreta spectrum, and placenta previa [15]. In an early study focused on cesarean delivery, factors including arrest disorders of labor, preeclampsia, and obesity were associated with the greatest risk of hemorrhage (estimated blood loss >1500 mL, ≥10% decrease in hematocrit, or packed red blood cell transfusion) [16]. In a secondary analysis of the Maternal-Fetal Medicine Units Network Cesarean Registry, a regression model incorporating variables at the time of cesarean delivery accurately predicted the need for transfusion (AUC, 0.82; 95% CI, 0.80, 0.84) [11]. The strongest predictors of transfusion were placenta previa and eclampsia/Hemolysis, elevated liver enzymes, and low platelets (HELLP) syndrome [11].

### 4.3 | Clinical implications

The current study and its findings may be generalized to most obstetrical populations. The cohort was large, multicentered, recruited prospectively, and accurately phenotyped for the parent clinical trial [13]. Patients underwent scheduled and unscheduled cesarean deliveries, and risk factors for red blood cell transfusion were easy to identify, included both antenatal and intrapartum variables (reflecting dynamic clinical scenarios during labor), and could be used in a standardized manner among all patients when considering crossmatch or other preparatory measures prior to cesarean delivery.

Potentially important observations in this study are the relationships between aspirin use in the week before delivery and preoperative anemia with increased incidence of red blood cell transfusion. As to the former, in the original trials published by the Maternal-Fetal Medicine Units Network studying low-dose aspirin to prevent preeclampsia, patients were instructed to continue low-dose aspirin until delivery, and rates of postpartum hemorrhage and transfusion were similar in the treatment and placebo groups [17, 18]. In a large national registry, self-reported aspirin use at any prenatal visit was associated with a higher incidence of postpartum hemorrhage, but after stratifying by mode of birth, a higher incidence of bleeding among aspirin users was present for those who had a vaginal birth but not those who had a cesarean delivery [19]. Two recent studies reported results in which low-dose aspirin was discontinued before delivery: (1) when low-dose aspirin was discontinued at 36 weeks, no increased risk of bleeding was observed when compared with patients not receiving aspirin [20], and (2) patients who discontinued low-dose aspirin <7 days before delivery had a higher risk of a postpartum hemorrhage composite outcome (estimated blood loss >1000 mL or red blood cell transfusion) compared to patients who discontinued low-dose aspirin ≥7 days before delivery (15.0% vs. 9.3%, unadjusted *p* = 0.03) [21]. Stratified analyses by mode of delivery were not performed [21]. Finally, in a recent systematic review and meta-analysis, the overall incidence of postpartum hemorrhage was 9.7% among patients who took low-dose aspirin during pregnancy compared with 7.9% among those who took a placebo or no treatment [22].

In this analysis, we found that there was an increased risk for postpartum hemorrhage among patients who used low-dose aspirin in the last week before cesarean delivery compared to patients who discontinued low-dose aspirin more than 1 week before cesarean delivery or never took low-dose aspirin during the pregnancy. This finding should not be considered causal in nature or indicate that low-dose aspirin should be discontinued more than a week prior to delivery—the risk for postpartum hemorrhage may have been driven by the clinical indications for the aspirin and characteristics of patients in this group rather than the aspirin itself.

A straightforward inference of our findings is that clinicians should identify and remediate anemia in the antepartum period with the intent of optimizing preoperative hemoglobin.

### 4.4 | Research implications

The current study identified four antenatal variables and three intrapartum variables that may be used clinically to identify patients at greatest risk of red blood cell transfusion, but additional work is needed to develop a calculator that automatically updates as part of the electronic medical record to determine individual patients’ risk for red blood cell transfusion at the onset of cesarean delivery. Implementation research may determine if use of a calculator leads to improved health outcomes.

### 4.5 | Strengths and limitations

Strengths of this study are that it was multicentered, contemporary, and included both scheduled and unscheduled cesarean deliveries. The primary treatment in the parent clinical trial (one-gram TXA intravenous immediately following umbilical cord clamping vs. placebo) did not affect rate of transfusion, so the entire large cohort (*N* = 10,995 patients) could be used for this secondary analysis. In addition, the transfusion outcome was carefully adjudicated by a panel of obstetric clinical investigators. The primary limitation is that the prediction model was not validated in an independent population, although internal validation was performed using *k*-fold cross-validation. In addition, this model cannot be used to know which interventions are optimal to prevent postpartum hemorrhage requiring transfusion.

## 5 | CONCLUSIONS

We identified several factors associated with risk of red blood cell transfusion during/after cesarean delivery and a model with excellent predictive ability. Some of the risk factors in the model, including low preoperative hemoglobin level, aspirin use <7 days before delivery, and delivery during the second stage of labor, may be used to risk-stratify patients when considering crossmatch or other preparatory measures prior to cesarean delivery to reduce postoperative complications that result from bleeding.

## Figures and Tables

**TABLE 1:** Factors considered for prediction model, comparing patients who received a transfusion and those who did not.

Characteristic	Transfused (*N* = 418)	Not transfused (*N* = 10,543)	*p* value
Twin pregnancy	35 (8.4)	421 (4.0)	<0.001
Non-spontaneous pregnancy (IVF/OI/AI)	29 (6.9)	487 (4.6)	0.03
Gestational age (weeks)			<0.001
<34	27 (6.5)	267 (2.5)	
34–36	57 (13.6)	1304 (12.4)	
37–40	311 (74.4)	8701 (82.5)	
≥41	23 (5.5)	270 (2.6)	
Age ≥40 years	31 (7.4)	554 (5.3)	0.05
Any cigarettes smoked in week before delivery	27 (6.5)	746 (7.1)	0.63
Liver disease	7 (1.7)	91 (0.9)	0.10
Diabetes			0.80
None	353 (84.5)	8803 (83.5)	
Type 1	4 (1.0)	138 (1.3)	
Type 2	14 (3.4)	434 (4.1)	
GDM	47 (11.2)	1168 (11.1)	
Neurologic disease	19 (4.5)	382 (3.6)	0.32
Cardiovascular disease	4 (1.0)	131 (1.2)	0.60
Respiratory disease	52 (12.4)	922 (8.8)	0.009
Renal disease	1 (0.2)	63 (0.6)	0.52
BMI (kg/m^2^)	35.7 ± 8.4	35.7 ± 8.2	0.81
Third trimester bleeding	22 (5.3)	413 (3.9)	0.16
Polyhydramnios	23 (5.5)	419 (4.0)	0.12
Hypertensive disorders			<0.001
None	272 (65.1)	7988 (75.8)	
Preeclampsia/gestational HTN	113 (27.0)	1810 (17.2)	
Chronic hypertension	33 (7.9)	745 (7.1)	
Chorioamnionitis	38 (9.1)	328 (3.1)	<0.001
Coagulopathy			0.63
Gestational thrombocytopenia	5 (1.2)	121 (1.1)	
Other	2 (0.5)	34 (0.3)	
Heparin prophylaxis at any time	4 (1.0)	51 (0.5)	0.16
Heparin prophylaxis in last day before delivery	3 (0.7)	35 (0.3)	0.18
Aspirin in the week before delivery	70 (16.8)	1155 (11.0)	<0.001
Preoperative hemoglobin	10.5 ± 1.6	11.7 ± 1.2	<0.001
Preoperative platelet count <100,000	8 (1.9)	63 (0.6)	0.006
Type of labor			<0.001
None	184 (44.0)	6767 (64.2)	
Spontaneous	66 (15.8)	1327 (12.6)	
Induced	168 (40.2)	2449 (23.2)	
Prostaglandin for cervical ripening	56 (13.4)	1007 (9.6)	0.009
Foley catheter for cervical ripening	131 (31.3)	1770 (16.8)	<0.001
Laminaria or Dilapan for cervical ripening	0 (0.0)	38 (0.4)	0.40
Oxytocin used	203 (48.6)	2778 (26.3)	<0.001
Oxytocin duration (h)	8.6 ± 11.1	4.3 ± 8.9	<0.001
Oxytocin maximum dose (mU/min)	8.9 ± 11.8	4.2 ± 8.7	<0.001
Complete cervical dilation	50 (12.0)	447 (4.2)	<0.001
Length of labor (h)	10.6 ± 13.2	5.4 ± 9.7	<0.001
Cesarean for labor arrest disorder	126 (30.1)	1600 (15.2)	<0.001
Magnesium sulfate used	56 (13.4)	816 (7.7)	<0.001
Prior vaginal deliveries			<0.001
None	291 (69.6)	8234 (78.1)	
1	62 (14.8)	1265 (12.0)	
2	34 (8.1)	624 (5.9)	
3	15 (3.6)	236 (2.2)	
>3	16 (3.8)	184 (1.7)	
Prior cesarean deliveries			<0.001
None	247 (59.1)	4567 (43.3)	
1	101 (24.2)	3574 (33.9)	
>1	70 (16.7)	2402 (22.8)	
TXA study arm	192 (45.9)	5314 (50.4)	0.07

*Note*: Data represent *N* (%) or mean ± standard deviation. Numbers of missing values are as follows: gestational age (*n* = 1), any cigarettes (*n* = 5), BMI (*n* = 33), third trimester bleeding (*n* = 5), aspirin (*n* = 43), preoperative hemoglobin (*n* = 8), preoperative platelet count (*n* = 170), oxytocin duration (*n* = 7), oxytocin dose (*n* = 1), and length of labor (*n* = 41).

Abbreviations: AI, artificial insemination; BMI, body mass index; GDM, gestational diabetes mellitus; HTN, hypertension; IVF, in vitro fertilization; OI, ovulation induction; TXA, tranexamic acid.

**TABLE 2:** Factors associated with transfusion and retained in the final prediction model.^a^

Characteristic	Transfused (*N* = 415)	Not transfused (*N* = 10,321)	Odds ratio	95% CI
Gestational age (weeks)				
<34	27 (6.5)	266 (2.6)	2.32	1.48, 3.63
34–36	56 (13.5)	1276 (12.4)	1.08	0.79, 1.47
37–40	309 (74.5)	8514 (82.5)	Ref	
≥41	23 (5.5)	265 (2.6)	2.06	1.28, 3.31
Aspirin in the week before delivery	69 (16.6)	1141 (11.1)	1.78	1.34, 2.37
Preoperative hemoglobin (g/dL)	10.5 ± 1.6	11.7 ± 1.2	0.45^b^	0.41, 0.48
Preoperative platelet count <100,000	8 (1.9)	63 (0.6)	3.73	1.69, 8.25
Chorioamnionitis	37 (8.9)	322 (3.1)	1.74	1.16, 2.61
Oxytocin duration (h)	8.6 ± 11.1	4.3 ± 8.9	1.04^b^	1.03, 1.05
Complete cervical dilation	50 (12.0)	431 (4.2)	2.74	1.93, 3.89

*Note*: Data represent *N* (%) or mean ± standard deviation, unless otherwise described.

Abbreviation: CI, confidence interval.

a Includes all observations with complete information for all characteristics in the model.

b Per one unit increase.

**TABLE 3:** Examples of individual predicted probabilities for transfusion based on the final prediction model.

Example	Gestational age (weeks)	Aspirin in the week before delivery	Preoperative hemoglobin (g/dL)	Preoperative platelet count <100,000	Chorioamnionitis	Oxytocin duration (h)	Cervix completely dilated	% Probability of transfusion (95% CI)
1	37–40	No	12.5	No	No	0	No	0.8 (0.6,1.0)
2	37–40	No	9.3	No	Yes	36	Yes	65.8 (54.1, 75.8)
3	37–40	No	10.0	No	No	0	No	5.9 (5.0, 6.9)
4	34–36	No	11.9	No	No	0	No	1.4 (1.0, 2.0)
5	34–36	Yes	11.0	No	No	0	No	5.1 (3.6, 7.1)
6	<34	Yes	10.5	No	Yes	18	No	36.5 (23.7, 51.5)
7	>41	Yes	9.0	Yes	Yes	24	Yes	95.6 (88.3, 98.4)

*Note*: Data represent *N* (%) or mean ± standard deviation, unless otherwise described.

Abbreviation: CI, confidence interval.

## Data Availability

The data that support the findings of this study are available from the corresponding author upon reasonable request.
